# Supplementary material for: Incentive Salience, not Psychomotor Sensitization or Tolerance, Drives Escalation of Cocaine Self-Administration in Heterogeneous Stock Rats
Source: bioRxiv. 2025 Nov 29:2025.10.04.680105. Preprint. [Version 3] doi: 10.1101/2025.10.04.680105 (PMC12667895; doi:10.1101/2025.10.04.680105)
Supplement: Supplement 2 [file media-2.pdf]

Mixed Effects Models Used in Analyses

Manuscript Plot	Mixed Model Type	Response Variable	Fixed Effects	Random Effects				Dispersion Modeling	Distribution Family (link)	AIC	BIC	Log-Likelihood	Deviance	Observations	DHARMa Plot
				Group	Effect	Std. Dev.	Variance								
Figure 1.B.	Generalized	Cocaine Infusions Short Access	Session	Rat	Intercept	1.42	1.19	Abstinence	nbinomal1 (log)	1940.9	1995.3	-956.4	1912.9	362	A
Figure 1.B.	Generalized	Cocaine Infusions Long Access	Session	Rat	Intercept	568.4	23.84	N/A	gaussian (identity)	4822	4889.6	-2395	4790	504	B
				Residual	-	651	25.51								
Figure 1.C.	Generalized	Cocaine Infusions First 15mins	Session	Rat	Intercept	0.59	0.77	Session	tweedie (log)	2669.6	2907.8	-1284.8	2569.6	866	C
Figure 2.A.	Linear	Locomotion Raw Noncontingent Sessions	Session	Rat	Intercept	0.09	0.29	N/A	gaussian (identity)	1140.9	1182.6	-559.5	1118.9	327	D
				Residual	-	1.72	1.31								
Figure 2.B.	Linear	Locomotion Percent Difference From Baseline Noncontingent Sessions	Session	Rat	Intercept	0.1	0.31	N/A	gaussian (identity)	369.6	396.4	-176.8	353.6	212	E
				Residual	-	0.26	0.51								
Figure 2.C.	Generalized	Locomotion Percent Difference From Drug 01	Session * Behavioral Expression	Rat	Intercept	0.13	0.35	Behavioral Expression	gaussian (identity)	187.4	203.2	-86.7	173.4	71	F
Figure 2.D.	Generalized	Cocaine Infusions Short Access	Session * Behavioral Expression	Rat	Intercept	1.46	1.21	Session	tweedie (log)	1966.9	2090.5	-951.4	1902.9	352	G
Figure 2.D.	Generalized	Cocaine Infusions Long Access	Session * Behavioral Expression	Rat	Intercept	580.6	24.1	Session * Behavioral Expression	gaussian (identity)	4706.2	4945.3	-2296.1	4592.2	490	H
Figure 2.E	Generalized	Cocaine Infusions First 15mins	Session * Behavioral Expression	Rat (Conditional)	Intercept	0.05	0.23	Session * Behavioral Expression + Rat	nbinomial12 (log)	2733.3	3202.2	-1267.7	2535.3	842	I
				Rat (Dispersion)	Intercept	7.3	2.7								
Figure 3.A	Generalized	Locomotion Pre-Lever	Session	Rat	Intercept	0.34	0.58	Abstinence	gaussian (identity)	2877	3010.2	-1410.5	2821	860	J
Figure 3.B.	Generalized	Locomotion Pre-Lever	Session Type * Abstinence	Rat	Intercept	0.32	0.57	Session Type * Abstinence* Infusion Group	gaussian (identity)	2875	2965.3	-1418.5	2837	860	K
Figure 3.H	Generalized	Active Lever Entrances per Meter Pre-Lever	Session	Rat (Conditional)	Intercept	0.15	0.39	Session Type * Abstinence + Rat	tweedie (log)	1475.3	1632.2	-704.6	1409.3	859	L
				Rat (Dispersion)	Intercept	0.11	0.34								
Figure 3.I.	Generalized	Active Lever Entrances per Meter Pre-Lever	Session Type * Abstinence	Rat (Conditional)	Intercept	0.14	0.38	Session + Rat	tweedie (log)	1514.7	1671.6	-724.3	1448.7	859	M
				Rat (Dispersion)	Intercept	0.13	0.37								
Figure 4.A.	Generalized	Lever Entrances per Meter Percent Difference From Noncontingent 01	Session	Rat (Conditional)	Intercept	0.83	0.91	Session + Rat	gaussian (identity)	731.1	755.1	-357.5	715.1	148	N
				Rat (Dispersion)	Intercept	0.17	0.42								
Figure 4.C.	Generalized	Active Lever Entrances per Meter Percent Difference From Noncontingnet 01	Session * Pre-Lever Activity	Rat (Conditional)	Intercept	0.11	0.33	Pre-Lever Activity + Rat	gaussian (identity)	361.5	379.9	-172.8	345.5	74	O
				Rat (Dispersion)	Intercept	0.54	0.74								
Figure 4.D.	Generalized	Cocaine Infusions Short Access	Session * Pre-Lever Activity	Rat	Intercept	1.27	1.13	Abstinence	tweedie (log)	1947.1	2044.4	-948.6	1897.1	362	P
Figure 4.D.	Generalized	Cocaine Infusions Long Access	Session * Pre-Lever Activity	Rat	Intercept	364.6	19.1	Pre-Lever Activity	gaussian (identity)	4807.3	4938.2	-2372.6	4745.3	504	Q
Figure 4.E.	Generalized	Cocaine Infusions First 15mins	Session * Pre-Lever Activity	Rat (Conditional)	Intercept	0.09	0.3	Session + Rat	nbinomial12 (log)	2737	3094.3	-1293.5	2587	866	R
				Rat (Dispersion)	Intercept	3.81	1.95								
Supplementary Figure 1.	Generalized	Nose Motion Percent Difference From Drug 01	Session * Behavioral Expression	Rat	Intercept	0.05	0.22	Session * Behavioral Expression	gaussian (identity)	87.2	109.9	-33.6	67.2	61	S
				Rat (Dispersion)	Intercept	3.26E-10	1.81E-05								
N/A	Generalized	Cocaine Infusions Short Access	Session * Sex	Rat	Intercept	1.45	1.21	Session	nbinomal1 (log)	1952.6	2073.2	-945.3	1890.6	331	T
N/A	Generalized	Cocaine Infusions Long Access	Session * Sex	Rat	Intercept	527.1	22.96	Session	gaussian (identity)	4829.1	5010.7	-2371.6	4743.1	461	U
N/A	Generalized	Locomotion Percent Difference From Baseline Noncontingent Sessions	Session * Sex	Rat	Intercept	0.1	0.31	Sex	gaussian (identity)	372.6	423	-171.3	342.6	197	V
N/A	Generalized	Cocaine Infusions Short Access	Session * Behavioral Expression * Sex	Rat	Intercept	1.53	1.24	Session * Sex	tweedie (log)	1978.7	2218.3	-927.4	1854.7	290	W
N/A	Generalized	Cocaine Infusions Long Access	Session * Behavioral Expression * Sex	Rat	Intercept	422.6	20.56	Abstinence * Sex	gaussian (identity)	4715.8	4980.1	-2294.9	4589.8	427	X
N/A	Generalized	Active Lever Entrances per Meter Pre-Lever	Session Type * Abstinence * Sex	Rat (Conditional)	Intercept	0.13	0.37	Session * Sex + Rat	tweedie (log)	1532.3	1831.9	-703.2	1406.3	796	Y
				Rat (Dispersion)	Intercept	0.14	0.37								
N/A	Generalized	Lever Entrances per Meter Percent Difference From Noncontingent 01	Session * Levers * Sex	Rat (Conditional)	Intercept	1.02	1.01	Session * Sex + Rat	gaussian (identity)	744.9	786.9	-358.5	716.9	134	Z
				Rat (Dispersion)	Intercept	0.11	0.33								
Supplementary Figure 2.	Generalized	Cocaine Infusions Short Access	Session * Pre-Lever Activity * Sex	Rat	Intercept	1.27	1.13	Pre-Lever Activity * Sex	tweedie (log)	1944.1	2123.2	-926.1	1852.1	316	AA
Supplementary Figure 2.	Linear	Cocaine Infusions Long Access	Session * Pre-Lever Activity * Sex	Rat	Intercept	290.2	17.04	N/A	gaussian (identity)	4811.3	5056.2	-2347.6	4695.3	446	BB
				Residual	-	558.3	23.63								
